# Yellow Nested Melanoma: Line‐Field Confocal Optical Coherence Tomography and Literature Review

**DOI:** 10.1111/ajd.14604

**Published:** 2025-09-26

**Authors:** Luca Bettolini, Vincenzo Maione, Andrea Carugno, Zeno Fratton, Enzo Errichetti, Mariachiara Arisi, Nicola Zerbinati, Iacopo Ghini, Stefano Bighetti

**Affiliations:** ^1^ Dermatology Department University of Brescia, ASST Spedali Civili di Brescia Brescia Italy; ^2^ Department of Medicine and Surgery University of Insubria Varese Italy; ^3^ Department of Medicine, Institute of Dermatology University of Udine Udine Italy

**Keywords:** dermopathology, LC‐OCT, melanoma, nested melanoma, non‐invasive diagnosis

## Abstract

Nested melanoma, a relatively new subtype of melanoma first reported in 2012, is characterised by neoplastic cells organised into nests. We present a unique case of yellow nested melanoma, detailing its clinical, dermoscopic, RCM, and, for the first time to our knowledge, LC‐OCT features. Alongside a comprehensive review of the literature, our findings challenge the traditional associations with advanced age and sun‐damaged skin, advocating for the term ‘nested melanoma’ to better reflect its characteristics.

Nested melanoma, first reported in the literature in 2012 [1], is a relatively new subtype of melanoma characterised by neoplastic cells organised into nests. Here, we present a new case of nested melanoma, detailing its clinical, dermoscopic, and reflectance confocal microscopy (RCM) features. For the first time to our knowledge, we also describe its characteristics using line‐field confocal optical coherence tomography (LC‐OCT), accompanied by a review of the existing literature.

A 75‐year‐old man with a history of pT2a N1 stage IIIA melanoma, diagnosed 6 months earlier, was referred to our melanoma outpatient clinic for his skin examination. He had declined adjuvant therapy, which had been offered according to protocol. During the visit, a 1 cm asymmetric, pigmented lesion was identified in the right supraclavicular region (Figure 1a). Dermoscopic evaluation revealed yellowish globules, occasionally confluent, sometimes containing central brownish dots, and shiny white structures (Figure 1b,c). RCM demonstrated round, bright cells corresponding to atypical cells within hyporeflective roundish nests arranged in a characteristic clod pattern (Figure 1d). LC‐OCT confirmed these findings, revealing round to oval bright atypical cells organised into nests of different dimensions, and surrounded by a dense inflammatory infiltrate characterised by small, hyper‐reflective cells in the papillary dermis (Figure 2a). In consideration of the findings from non‐invasive diagnostic tools, the lesion was excised. Histopathology (Figure 2b–d) revealed a predominantly junctional nevic lesion, characterised by variably sized nests forming a distinct ‘nested’ pattern. The lesion also exhibited a junctional component with single melanocytic cells demonstrating a tendency for vertical suprabasal growth, highly heterogeneous nests, and a rich inflammatory infiltrate unevenly distributed across the lesion. Immunohistochemistry highlighted the nested organisation of MART1+ cells, while intense and diffuse PRAME positivity confirmed the malignant nature of the lesion. Mild solar elastosis, focal pagetoid spread, and a common acquired nevus associated with melanoma were observed. A diagnosis of nested melanoma with a Breslow thickness (BT) of 0.6 mm was established.

**FIGURE 1:**
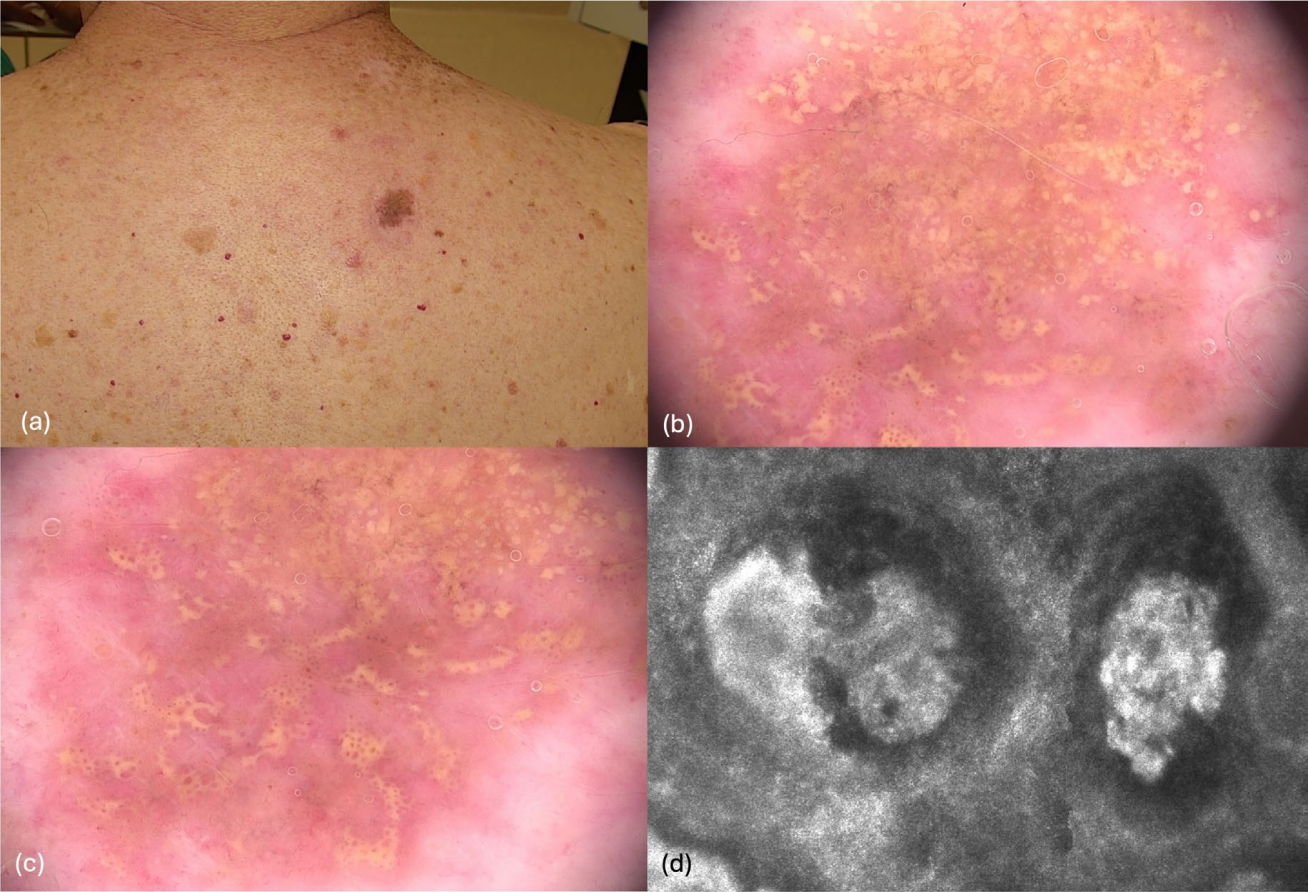
(a) 1 cm pigmented lesion on the back. (b, c) Dermoscopy revealed yellowish globules, occasionally confluent and sometimes containing central brownish dots, along with shiny white structures. (d) Reflectance Confocal Microscopy (RCM) demonstrated round, bright cells within hyporeflective roundish nests.

**FIGURE 2:**
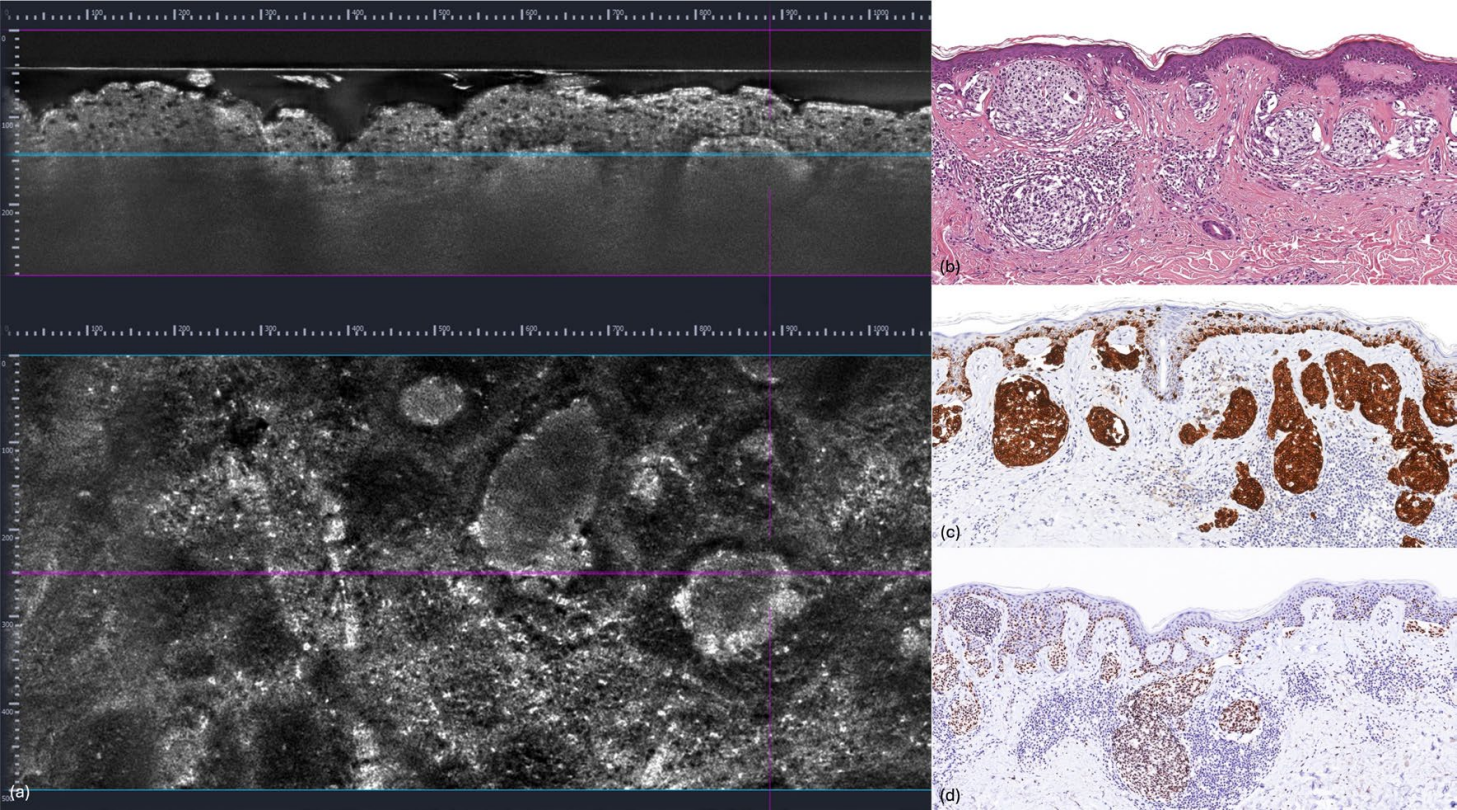
(a) Line‐field confocal optical coherence tomography (LC‐OCT) revealed round‐to‐oval bright atypical cells organised into nests of varying sizes, surrounded by a dense inflammatory infiltrate composed of small, hyper‐reflective cells in the papillary dermis. (b) Histopathological exam revealing a nevic lesion, predominantly junctional, characterised by variably sized nests that form a “nested” pattern. Additionally, the lesion displays a junctional component with single cells showing a tendency for vertical suprabasal growth, highly heterogeneous nests, and a rich inflammatory infiltrate unevenly distributed throughout the lesion (H&E, magnification 40×). (c) Immunohistochemistry highlighting the “nested” organisation of the MART1+ cell population (MART1, magnification 40×). (d) The intense and diffuse PRAME positivity further substantiates the malignant nature of the lesion (PRAME, magnification 40×).

Including our case, 38 cases of nested melanoma have been reported to date [1, 2, 3, 4, 5, 6, 7] (Table 1). Among them, 20 patients (52.6%) were female, and 18 (47.4%) were male. The median age at diagnosis was 63 years (IQR: 29–90 years). Previously referred to as ‘nested melanoma of the elderly,’ data reveal that 15 (39.5%) of the patients were under 60 years old, and notably, 5 (13.2%) were under 40 years old, redefining its association with advanced age. The lesions were distributed across different body sites: 12 (31.6%) on the lower limbs, 10 (26.3%) on the upper limbs, 9 (23.7%) on the back, and 7 (18.4%) on the trunk. The diameter of the lesions varied widely, ranging from 6 to over 30 mm. When clinically described, the lesions appeared irregular in shape and colour. Dermoscopically, the lesions were predominantly characterised by irregular dots and globules. Notably, only our patient exhibited a yellow pattern surrounding the globules. This feature appears to be attributable to a dense inflammatory infiltrate, consistent with histopathological findings and previously hypothesised in other conditions [8]. Of the 37 documented BT cases, 20 (54%) were in situ melanomas, 15 (40.6%) had a BT of less than 7 mm, and 2 (5.4%) had a BT exceeding 8 mm. Histological analysis (Table S1) revealed that 18 lesions (47.4%) were circumscribed, while 4 (10.5%) lacked circumscription. Pagetoid spread was identified in 17 cases (44.7%), whereas 7 cases (18.4%) showed no such pattern. Dermal invasion was observed in 18 lesions (47.4%), with the remaining 20 lesions (52.6%) confined to the epidermis. Lentiginous growth was present in 12 cases (31.6%), with 1 case (2.6%) classified as uncertain. None of the 22 evaluated cases exhibited epidermal consumption. In terms of cellular atypia, 10 cases (26.3%) demonstrated mild atypia, 11 (28.9%) moderate atypia, another 11 (28.9%) moderate to severe atypia, 3 cases (7.9%) showed severe atypia, and for 3 cases (7.9%), data were unavailable. In 26 cases (68.4%), nests were surrounded by a rim of keratinocytes, while 1 case (2.6%) did not exhibit this feature. Solar elastosis was absent in 8 cases (21.1%), mild in 6 cases (15.8%), moderate in 8 cases (21.1%), and severe in 1 case (2.6%). In the remaining 11 cases (28.9%) described by Kutzner et al., solar elastosis was reported inconsistently, with varying degrees. The term ‘nested melanoma in sun‐damaged skin’ was introduced as a more accurate alternative to ‘nested melanoma of the elderly’. However, it is important to note that solar elastosis is absent in at least 20% of cases. Of the 14 cases reported, 7 (50%) had a nevus associated with melanoma, while the remaining 7 (50%) did not. p16 immunostaining was positive in 4 cases (28.6%) and negative in 2 cases (14.3%), whereas BRAF V600E mutation analysis showed positivity in 6 cases (15.8%) and negativity in 9 cases (23.7%). LC‐OCT is a non‐invasive diagnostic tool capable of examining cutaneous tissue up to a depth of 500 μm, providing a deeper view compared to routine RCM, which only reaches the upper dermis [9]. By extending into the superficial dermis, LC‐OCT enables precise identification and analysis of nested patterns and cellular atypia. Dermoscopy, although characterised by an unusual yellow pattern, did not reveal definitive features or patterns suggestive of melanoma. However, LC‐OCT proved instrumental in raising clinical suspicion.

**TABLE 1:** Summary of the clinical features of nested melanomas reported in the literature.

Case		Age	Sex	Body site	Clinical	Dermosocpy	RCM	LC‐OCT	Stage
Current		75	M	Back	10 × 10 mm, asymmetric	Yellowish globules, occasionally confluent, sometimes containing central brownish dots. Shiny white structures.	Round, bright cells within hyporeflective roundish nests.	Round‐to‐oval bright atypical cells organised into nests of varying sizes, surrounded by a dense inflammatory infiltrate composed of small, hyper‐reflective cells in the papillary dermis.	BT 0.6 (pt1a)
2	Zardo et al. (2021) [7]	29	F	Calf	Asymmetric pigmentation	NA	NA	NA	BT 0.6 (pt1a)
3	Leecy et al. (2020) [6]	58	M	Deltoid	8 × 8 mm	NA	NA	NA	BT 0.7
4		69	M	Shoulder	11 × 11 mm	NA	NA	NA	BT 0.3
5		29	M	Deltoid	6 × 6 mm	NA	NA	NA	BT 0.5
6		58	F	Thigh/hip	11 × 11 mm	NA	NA	NA	BT 0.3
7		31	M	Leg	12 × 12 mm	NA	NA	NA	pTis
8		39	M	Back	12 × 12 mm	NA	NA	NA	BT 1.1
9		47	M	Chest wall	7 × 7 mm	NA	NA	NA	NA
10		69	M	Back	4 × 4 mm	NA	NA	NA	pTis
11		83	M	Upper back	14 × 14 mm	NA	NA	NA	pTis
12		71	F	Back	12 × 12 mm	NA	NA	NA	pTis
13		60	M	Thigh	6 × 6 mm	NA	NA	NA	BT 0.5
14		68	M	Back	8 × 8 mm	NA	NA	NA	BT > 0.6
15	Jegou et al. (2017) [5]	52	M	Right chest wall	10 × 10 mm, irregular and multicoloured	Asymmetric, featuring an off‐center blackish, structureless ink‐blot area with segmented radial streaks and peripheral brown and black globules.	NA	NA	BT 0.6 (pt1a)
16	Casari et al. (2014) [4]	70	F	Left leg	9 × 8 mm, irregular shape	Asymmetrical, predominantly globular pattern with remnants of a melanocytic network. Brown globules of varying size and colour (light brown to black) were irregularly distributed.	NA	NA	pTis
17	Longo et al. (2013) [1]	65	F	Back	14 × 14 mm, irregular shape and colour	Asymmetric, with a predominant globular pattern featuring pigmented globules of varying size and colour.	Clod pattern (dense nests arranged in clusters) observed at the dermo‐epidermal junction, composed of polymorphic cells distorting the interpapillary spaces. Cytological atypia were identified within the nests, with focal pagetoid cells present in the basal layers of the epidermis.	NA	pTis
18		70	F	Right calf	25 × 20 mm, variegated	Predominant globular pattern with pigmented globules exhibiting striking variation in colour and distribution throughout the lesion.	Clod pattern at the dermoepidermal junction composed of regularly spaced large compact nests. Cytologic atypia was found within the nests.	NA	BT 0.4
19		50	M	Calf	6 × 6 mm, irregularly pigmented	Asymmetric in colour and structure, featuring irregularly distributed dots and globules on a structureless background ranging from light to dark brown.	Predominant clod pattern with large nests, accompanied by focal pagetoid spread and cytologic atypia at the dermo‐epidermal junction.	NA	pTis
20	Pennacchia et al. (2012) [3]	90	F	Four on the trunk, three on the lower extremities, and one on the forearm.	10 × 10 mm	NA	NA	NA	pTis
21		61	F		7 × 7 mm	NA	NA	NA	pTis
22		37	F		8 × 8 mm	NA	NA	NA	BT 0.3
23		49	F		12 × 12 mm	NA	NA	NA	pTis
24		67	F		> 30 mm	NA	NA	NA	pTis
25		76	F		10 × 10 mm	NA	NA	NA	pTis
26		80	F		6 × 6 mm	NA	NA	NA	pTis
27		65	F		10 × 10 mm	NA	NA	NA	pTis
28	Kutzner et al. (2012) [2]	49	F	Upper arm	> 6 mm diameter, asymmetry of colour and shape, irregular borders, multiple colours.	Asymmetry with a multicomponent structure, irregular blotches, atypical pigment network, and large, irregular round‐to‐oval dots and globules.	NA	NA	pTis
29		70	F	Back			NA	NA	pTis
30		71	M	Upper arm			NA	NA	pTis
31		74	M	Shoulder			NA	NA	BT 0.3
32		61	M	Forearm			NA	NA	BT 1.5
33		61	M	Forearm			NA	NA	BT 0.3
34		56	F	Upper arm			NA	NA	BT 0.5
35		44	F	Upper back			NA	NA	BT 0.3
36		79	F	Foot			NA	NA	pTis
37		69	M	Lower leg			NA	NA	pTis
38		54	F	Abdomen			NA	NA	pTis

Abbreviations: BT, breslow thickness; LC‐OCT, line‐field confocal optical coherence tomography; NA, not available; pTis, in situ melanoma; RCM, recflectante confocal microscopy.

In conclusion, we report a case of yellow nested melanoma, highlighting its clinical, dermoscopic, and RCM features. For the first time, to our knowledge, we also present the LC‐OCT features of nested melanoma. By reviewing the existing literature, the terms nested melanoma ‘of the elderly’ or ‘in sun‐damaged skin’ are misleading, as this entity can also occur in younger patients and in the absence of significant solar elastosis. It should simply be referred to as ‘nested melanoma’ to more accurately reflect its pathological characteristics.

## Consent

The patient in this manuscript has given written informed consent to publication of his case details.

## Conflicts of Interest

The authors declare no conflicts of interest.

## Supporting information


**Table S1:** Summary of histological features of nested melanomas reported in the literature.

## Data Availability

The data that support the findings of this study are available from the corresponding author upon reasonable request.
