# Supplementary material for: Yellow Nested Melanoma: Line‐Field Confocal Optical Coherence Tomography and Literature Review
Source: Australas J Dermatol. 2025 Sep 26;66(8):479–85. doi: 10.1111/ajd.14604 (PMC12687715; doi:10.1111/ajd.14604)
Supplement: Supplementary file 1 — Table S1: Summary of histological features of nested melanomas reported in the literature. [file AJD-66-479-s001.docx]

Supplemental table 1. Summary of histological features of nested melanomas reported in the literature.

| Case no. | Circumscription | Pagetoid spread | Dermal invasion | Lentiginous growth | Epidermal consumption | Atypia | Rim of basal keratinocytes | Solar elastosis | Co-existing lesion | P16 Immunostaining | BRAF V600E mutation |
| --- | --- | --- | --- | --- | --- | --- | --- | --- | --- | --- | --- |
| Current | Yes | Focal | Present | Present | Absent | Severe | Absent | Mild | Common acquired naevus | NA | NA |
| 2 | No | Focal | Present | NA | Absent | Mild | NA | Mild | NA | NA | Positive |
| 3 | Yes | Present | Present | Absent | Absent | Moderate | Present | Moderate | Intradermal naevus | Positive | NA |
| 4 | Yes | Absent | Present | Absent | Absent | Mild | Present | Moderate | Intradermal naevus | Positive | Negative |
| 5 | Yes | Present | Present | Absent | Absent | Mild | Present | Absent | Nil | Positive | Negative |
| 6 | No | Present | Present | Present | Absent | Moderate | Present | Absent | Nil | Negative | Positive |
| 7 | Yes | Present | Absent | Absent | Absent | Moderate | Present | Absent | Nil | Negative | Positive |
| 8 | Yes | Absent | Present | Absent | Absent | Moderate | Present | Absent | Intradermal naevus | Positive | Negative |
| 9 | Yes | Absent | Present | Absent | Absent | Moderate | Present | Absent | Nil | NA | NA |
| 10 | No | Absent | Absent | Absent | Absent | Moderate | Present | Moderate | Intradermal naevus | NA | Negative |
| 11 | No | Absent | Absent | Present | Absent | Moderate | Present | Moderate | Nil | NA | NA |
| 12 | Yes | Absent | Absent | Absent | Absent | Moderate | Present | Mild | Nil | NA | NA |
| 13 | Yes | Present | Present | Absent | Absent | Moderate | Present | Absent | Nil | NA | NA |
| 14 | Yes | Absent | Present | Absent | Absent | Mild | Present | Absent | Intradermal naevus | NA | NA |
| 15 | NA | NA | Absent | NA | NA | NA | Present | NA | NA | NA | NA |
| 16 | NA | Present | Absent | NA | NA | Focal | NA | NA | NA | NA | NA |
| 17 | NA | Focal | Present | NA | NA | Mild | NA | Mild | Present, NS | NA | NA |
| 18 | NA | NA | Present | NA | NA | NA | NA | NA | NA | NA | NA |
| 19 | NA | NA | Absent | NA | NA | NA | NA | NA | NA | NA | NA |
| 20 | Yes | Present | Absent | Present | Absent | Moderate | NA | Moderate | NA | NA | Positive |
| 21 | Yes | Present | Absent | Present | Absent | Mild | NA | Moderate | NA | NA | Positive |
| 22 | Yes | Present | Present | Absent | Absent | Mild | NA | Mild | NA | NA | Negative |
| 23 | Yes | Present | Absent | Absent | Absent | Mild | NA | Absent | NA | NA | Negative |
| 24 | Yes | Present | Absent | Present | Absent | Severe | NA | Mild | NA | NA | Negative |
| 25 | Yes | Present | Absent | Doubt | Absent | Mild | NA | Severe | NA | NA | Negative |
| 26 | Yes | Present | Absent | Present | Absent | Moderate | NA | Moderate | NA | NA | Positive |
| 27 | Yes | Present | Absent | Present | Absent | Severe | NA | Moderate | NA | NA | Negative |
| 28 | NA | NA | Absent | 4 case present, 7 cases absent | NA | Moderate to severe | Present | Not all, varying degress | NA | NA | NA |
| 29 | NA | NA | Absent |  | NA |  |  |  | NA | NA | NA |
| 30 | NA | NA | Absent |  | NA |  |  |  | NA | NA | NA |
| 31 | NA | NA | Present |  | NA |  |  |  | NA | NA | NA |
| 32 | NA | NA | Present |  | NA |  |  |  | NA | NA | NA |
| 33 | NA | NA | Present |  | NA |  |  |  | NA | NA | NA |
| 34 | NA | NA | Present |  | NA |  |  |  | NA | NA | NA |
| 35 | NA | NA | Present |  | NA |  |  |  | NA | NA | NA |
| 36 | NA | NA | Absent |  | NA |  |  |  | NA | NA | NA |
| 37 | NA | NA | Absent |  | NA |  |  |  | NA | NA | NA |
| 38 | NA | NA | Absent |  | NA |  |  |  | NA | NA | NA |
| NA: not available; NS: not specified; | | | | | | | | | | | |
